# Crystal‐Phase‐Selective Etching of Heterophase Au Nanostructures

**DOI:** 10.1002/smtd.202400430

**Published:** 2024-07-06

**Authors:** Faisal Saleem, Guangyao Liu, Guigao Liu, Bo Chen, Qinbai Yun, Yiyao Ge, An Zhang, Xixi Wang, Xichen Zhou, Gang Wang, Lingwen Liao, Zhen He, Lujiang Li, Hua Zhang

**Affiliations:** ^1^ Department of Chemistry City University of Hong Kong Hong Kong China; ^2^ Key Laboratory of Flexible Electronics (KLOFE) & Institute of Advanced Materials (IAM) & School of Flexible Electronics (Future Technologies) Nanjing Tech University Nanjing 211816 China; ^3^ National Special Superfine Powder Engineering Research Center School of Chemistry and Chemical Engineering Nanjing University of Science and Technology Nanjing 210094 China; ^4^ State Key Laboratory of Organic Electronics and Information Displays & Jiangsu Key Laboratory for Biosensors Institute of Advanced Materials Nanjing University of Posts and Telecommunications Nanjing 210023 China; ^5^ Department of Chemistry The Chinese University of Hong Kong Hong Kong China; ^6^ Key Laboratory of Materials Physics Anhui Key Laboratory of Nanomaterials and Nanotechnology Institute of Solid State Physics Chinese Academy of Sciences Hefei 230031 China; ^7^ Hong Kong Institute for Clean Energy City University of Hong Kong Kowloon Hong Kong China; ^8^ Hong Kong Branch of National Precious Metals Material Engineering Research Center (NPMM) City University of Hong Kong Hong Kong China; ^9^ Shenzhen Research Institute City University of Hong Kong Shenzhen 518057 China

**Keywords:** Au nanostructures, crystal phase engineering, heterophase, selective etching

## Abstract

Selective oxidative etching is one of the most effective ways to prepare hollow nanostructures and nanocrystals with specific exposed facets. The mechanism of selective etching in noble metal nanostructures mainly relies on the different reactivity of metal components and the distinct surface energy of multimetallic nanostructures. Recently, phase engineering of nanomaterials (PEN) offers new opportunities for the preparation of unique heterostructures, including heterophase nanostructures. However, the synthesis of hollow multimetallic nanostructures based on crystal‐phase‐selective etching has been rarely studied. Here, a crystal‐phase‐selective etching method is reported to selectively etch the unconventional 4H and 2H phases in the heterophase Au nanostructures. Due to the coating of Pt‐based alloy and the crystal‐phase‐selective etching of 4H‐Au in 4H/face‐centered cubic (*fcc*) Au nanowires, the well‐defined ladder‐like Au@PtAg nanoframes are prepared. In addition, the 2H‐Au in the *fcc*‐2H‐*fcc* Au nanorods and 2H/*fcc* Au nanosheets can also be selectively etched using the same method. As a proof‐of‐concept application, the ladder‐like Au@PtAg nanoframes are used for the electrocatalytic hydrogen evolution reaction (HER) in acidic media, showing excellent performance that is comparable to the commercial Pt/C catalyst.

## Graphical abstract

Unconventional‐phase 4H‐Au and 2H‐Au in heterophase Au nanostructures are selectively etched. The PtAg alloy coating and crystal‐phase‐selective etching of 4H‐Au in the 4H/*fcc* Au nanowires produce well‐defined ladder‐like Au@PtAg nanoframes. This method can also be used to etch 2H‐Au in the *fcc*‐2H‐*fcc* Au nanorods and 2H/*fcc* Au nanosheets, highlighting the importance of crystal phase in the etching of Au nanomaterials.

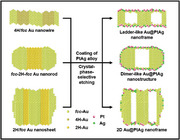

## 1 Introduction

Controlled synthesis of noble multimetallic nanomaterials is highly desirable because of their unique optical, electronic, and catalytic properties.^[^ ^1^, ^2^, ^3^, ^4^ ^]^ In the past decades, noble multimetallic nanocrystals with different architectures have been prepared through a variety of synthetic approaches.^[^ ^5^, ^6^, ^7^, ^8^, ^9^ ^]^ The etching process, as one of the typical top‐down methods, has been demonstrated as an attractive route to reshape the morphology of noble metal nanostructures.^[^ ^10^ ^]^ The mechanism of the etching process in multimetallic nanostructures normally depends on the different reactivity of metal components^[^ ^11^, ^12^, ^13^, ^14^ ^]^ and the distinct surface energy of different facets.^[^ ^15^, ^16^, ^17^ ^]^ For example, hollow nanostructures, such as hollow nanotubes, nanocages and nanoframes, have been prepared by the selective etching of more active metal cores in the core‐shell nanostructures.^[^ ^18^, ^19^, ^20^ ^]^ Similarly, facet‐selective etching, in which the surface atoms with higher surface energy are more susceptible to oxidation, can convert the primary nanostructures into complicated nanostructures with specific exposed facets.^[^ ^21^ ^]^ However, to date, the role of crystal phase, one of the most essential structural parameters which can affect the reactivity in etching process,^[^ ^22^ ^]^ has rarely been explored in the etching process of nanomaterials.

Recently, phase engineering of nanomaterials (PEN) has been proposed to demonstrate the importance of phase control in the preparation of nanomaterials with unique properties and functions.^[^ ^23^, ^24^, ^25^, ^26^ ^]^ For instance, our group has developed various wet‐chemical methods to synthesize a series of unconventional phase Au nanomaterials with hexagonal crystal structure (e.g., 2H‐Au nanosheets (NSs)^[^ ^27^ ^]^ and 4H‐Au nanoribbons)^[^ ^28^ ^]^ and heterophase Au nanostructures (e.g., 4H/face‐centered cubic (*fcc*) Au nanowires (NWs),^[^ ^29^ ^]^ *fcc*‐2H‐*fcc* Au nanorods (NRs)^[^ ^30^ ^]^ and 2H/*fcc* Au NSs).^[^ ^31^ ^]^ By using as‐synthesized 4H/*fcc* Au NWs, *fcc*‐2H‐*fcc* Au NRs and 2H/*fcc* Au NSs as templates, the significant effect of the crystal phase on the nucleation and epitaxial growth of nanostructures has been studied.^[^ ^29^, ^31^, ^32^ ^]^ However, the role of the crystal phase in the selective etching of nanostructures has not been investigated. Due to the difference in stability between the thermodynamically stable phase and unconventional metastable phase, it is feasible to selectively etch the Au with metastable 2H and/or 4H phases from the aforementioned heterophase Au nanomaterials to precisely construct nanostructures with well‐defined architectures for promising applications. Moreover, selectively etching the unconventional metastable phases from heterophase nanostructures while preserving the original framework in stable phases can potentially expose specific phases and increase the number of active sites.

Here, we propose a new wet‐chemical strategy for the controlled etching of our pre‐synthesized heterophase 4H/*fcc* Au NWs, *fcc*‐2H‐*fcc* Au NRs and 2H/*fcc* Au NSs. Our results show that the unconventional metastable phases, i.e., 4H and 2H phases, in the aforementioned heterophase Au nanomaterials can be selectively etched away. Impressively, various hollow Au@PtAg nanostructures, e.g., ladder‐like, dimer‐like, and sheet‐like Au@PtAg nanoframes, have been prepared. As a proof‐of‐concept application, when used as the catalyst for electrochemical hydrogen evolution reaction (HER) in acidic media, the ladder‐like Au@PtAg nanoframes show a comparable HER performance to the commercial Pt/C catalyst, requiring a low overpotential of −16.4 mV to achieve the current density of 10 mA cm^−2^.

## 2 Results and Discussion

**Figure 1:**
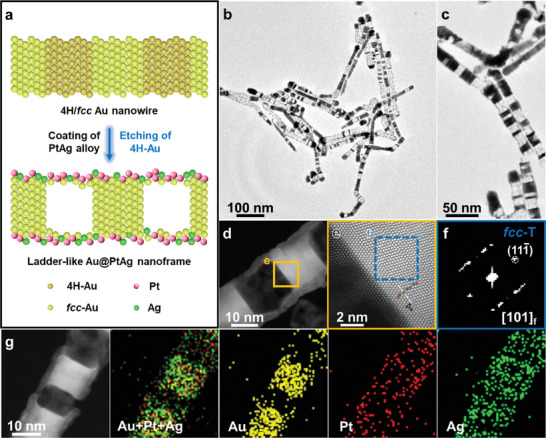
Crystal‐phase‐selective etching of 4H/*fcc* Au NWs. a) Schematic illustration of the crystal‐phase‐selective etching of 4H/*fcc* Au NW to form the ladder‐like Au@PtAg nanoframes. b,c) Low‐magnification TEM image (b) and high‐magnification TEM image (c) of the obtained ladder‐like Au@PtAg nanoframes. d) Aberration‐corrected HAADF‐STEM image of a representative ladder‐like Au@PtAg nanoframe. e) High‐resolution aberration‐corrected HAADF‐STEM image taken from the yellow square in d. The red, green, and blue balls represent the atoms in the close‐packed planes of the *fcc* phase with a stacking sequence of “ABC”. Twin boundary (T) is marked using the white dashed line. f) FFT pattern along the [101]_f_ zone axis taken from the dashed blue square in e. g) HAADF‐STEM image and corresponding EDS elemental mappings of a representative ladder‐like Au@PtAg nanoframe.

The crystal‐phase‐selective etching of heterophase Au nanostructures to obtain ladder‐like Au@PtAg nanoframes can be achieved via a wet‐chemical strategy (**Figure** **1**a; Figure S1, see details in Supporting Information). The preparation of Au@PtAg nanoframes involves two reactions, i.e., the deposition of Pt and Ag, and the etching of Au. When the synthesized 4H/*fcc* heterophase Au NWs (Figure S2, Supporting Information) served as seeds for the oxidative etching, the nanoframes with ladder‐like hollow structures were obtained (Figure 1b,c). As shown in Figure 1d, the aberration‐corrected high‐angle annular dark‐field scanning transmission electron microscopy (HAADF‐STEM) verifies the formation of hollow structures. The magnified HAADF‐STEM image (Figure 1e) and the corresponding fast Fourier transform (FFT) pattern (Figure 1f) demonstrate that thermodynamically stable *fcc*‐Au domains with twin boundaries remain after the etching process, indicating the selective etching of metastable 4H‐Au in the 4H/*fcc* heterophase Au NWs. Moreover, as shown in the X‐ray diffraction (XRD) patterns (Figure S3, Supporting Information), the weak intensities of typical peaks assigned to the 4H phase in the ladder‐like Au@PtAg nanoframes compared to the original 4H/*fcc* Au NWs further suggest the crystal‐phase‐selective etching of unconventional 4H‐Au domains. The HAADF‐STEM image and the corresponding elemental mapping results (Figure 1g) reveal the distributions of Au, Pt, and Ag elements, indicating the occurrence of the coating of Pt and Ag, and Au etching during the reactions. The formation of hollow ladder‐like Au@PtAg nanoframes can be ascribed to the selective etching of unconventional metastable 4H‐Au and the residue of thermodynamically stable *fcc*‐Au during the oxidative etching process, along with the deposition of homogeneous Pt and Ag alloys.

To investigate the effect of the amount of etchant solution on the selective etching of Au, a control experiment was carried out by following the same protocol except changing the amount of etchant solution from 0.05 mL to 4 mL. As indicated by the TEM results in Figure S4 (Supporting Information), no apparent selective etching of Au was observed, while the core‐shell Au@PtAg nanorods were prepared. Therefore, the usage of a small amount of etchant solution, i.e., 0.05 mL in our experiments, plays an important role in achieving the crystal‐phase‐selective etching of heterophase Au nanomaterials.

**Figure 2:**
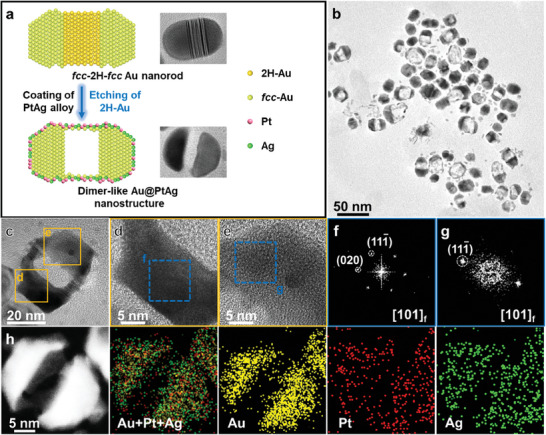
Crystal‐phase‐selective etching of *fcc*‐2H‐*fcc* Au NRs. a) Schematic illustration of the crystal‐phase‐selective etching of *fcc*‐2H‐*fcc* Au NR to form dimer‐like Au@PtAg hollow nanoframes and the corresponding TEM images. b) TEM image of the obtained dimer‐like Au@PtAg nanoframes. c) HRTEM image of a typical Au@PtAg dimer‐like nanoframe. d,e) Magnified HRTEM images taken from the corresponding squares in c. f,g) FFT patterns taken from the blue dashed squares in d and e, respectively. h) HAADF‐STEM image and corresponding EDS elemental mappings of a representative dimer‐like Au@PtAg nanoframe.

To further demonstrate the generality of such crystal‐phase‐selective etching process toward the preparation of novel hollow heterostructures, other heterophase Au nanomaterials, including the *fcc*‐2H‐*fcc* heterophase Au NRs and 2H/*fcc* heterophase Au NSs, have also been used for the etching experiments under the same conditions. As shown in Figure S5 (Supporting Information), the *fcc*‐2H‐*fcc* heterophase Au NRs with the *fcc* phase at two ends and the 2H phase in the middle were synthesized based on our previously reported method.^[^ ^30^ ^]^ As schematically shown in **Figure** **2**a, a unique dimer‐like hollow nanoframe is obtained after the selective etching process. The TEM image in Figure 2b shows the dimer‐like architectures of the as‐prepared nanoframes with a hollow area in the middle, suggesting the occurrence of crystal‐phase‐selective etching of 2H‐Au. A typical dimer‐like nanoframe (Figure 2c) is chosen to analyze the crystal phase of the etched product. The magnified HRTEM images (Figure 2d,e) and the corresponding FFT patterns (Figure 2f,g) show that the *fcc*‐Au domains still maintained after the etching process, further demonstrating the crystal‐phase‐selective etching of 2H‐Au. The HAADF‐STEM image and the corresponding EDS elemental mappings in Figure 2h reveal the distributions of the Au, Pt, and Ag elements, verifying the existence of Pt and Ag on the dimer‐like Au@PtAg nanoframe.

**Figure 3:**
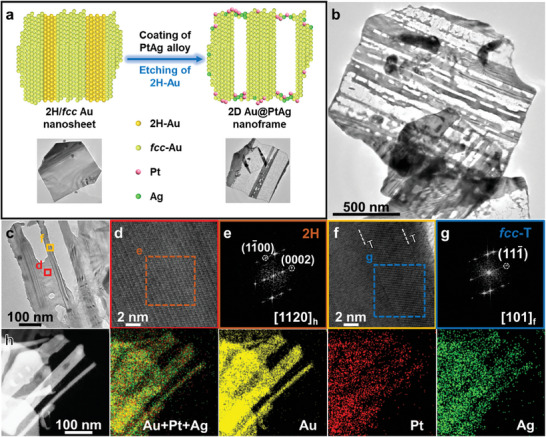
Crystal‐phase‐selective etching of 2H/*fcc* Au NSs. a) Schematic illustration of the crystal‐phase‐selective etching of 2H/*fcc* Au NS to form 2D Au@PtAg nanoframe and the corresponding TEM images. b) TEM image of the obtained 2D Au@PtAg nanoframes. c) TEM image of a partially etched 2H/*fcc* Au NS. d) HRTEM image taken from the red square in c. e) The corresponding FFT pattern taken from the orange dashed square in d. f) HRTEM image taken from the yellow square in c. Twin boundaries are marked with the white dashed lines. g) The corresponding FFT pattern taken from the blue dashed square in f. h) HAADF‐STEM image and EDS elemental mappings of the prepared 2D Au@PtAg nanoframes.

Similarly, the 2H/*fcc* heterophase Au NSs (Figure S6, Supporting Information), synthesized according to our previously reported method,^[^ ^31^ ^]^ were further used to prove the crystal‐phase‐selective etching under the same experimental conditions (**Figure** **3**a). The TEM image in Figure 3b shows the obtained nanostructures with hollow sheet‐like morphology, indicating the successful etching of Au. The hollow structure of the 2D Au@PtAg nanoframes was shown in Figure 3c. To further investigate the phase structure of the hollow 2D Au@PtAg nanoframes, the HRTEM images taken from different areas of the nanosheets in Figure 3c were systematically analyzed. As shown in Figure 3d, the non‐etched domain taken from the red square in Figure 3c possesses the 2H phase, as confirmed by the corresponding FFT pattern (Figure 3e). The HRTEM image of a remaining domain after etching (Figure 3f) and the corresponded FFT pattern (Figure 3g) show the *fcc* phase with twin boundaries along the close‐packed [111]_f_ direction. Compared to the original structure of 2H/*fcc* Au NSs, it can be found that 2H‐Au has been selectively etched while the *fcc*‐Au was maintained. The HAADF‐STEM image and EDS elemental mappings (Figure 3h) reveal the distributions of the Au, Pt, and Ag elements, indicating the deposition of Pt and Ag during the partial etching of 2H‐Au.

**Figure 4:**
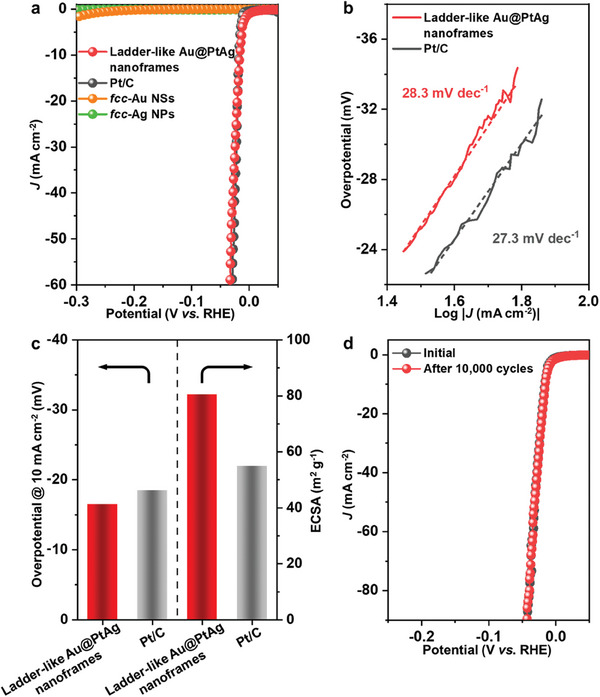
HER performances of different electrocatalysts. a) Polarization curves of the ladder‐like Au@PtAg nanoframes, *fcc*‐Ag NPs, *fcc*‐Au NSs, and the commercial Pt/C catalyst, which were recorded in a N_2_‐saturated 0.5 M H_2_SO_4_ aqueous solution at a scan rate of 5.0 mV s^−1^. b) Tafel slopes of the ladder‐like Au@PtAg nanoframes and Pt/C obtained from the polarization curves in a. c) Overpotentials at the current density of 10 mA cm^−2^ (left) and ECSAs (right) of the ladder‐like Au@PtAg nanoframes and Pt/C. d) Durability test of the ladder‐like Au@PtAg nanoframes. The polarization curves were recorded before and after 10000 potential cycles in a N_2_‐saturated 0.5 M H_2_SO_4_ aqueous solution.

As a proof‐of‐concept application, the obtained ladder‐like Au@PtAg nanoframes (Figure 1) were used as a catalyst for the electrochemical HER in 0.5 M H_2_SO_4_ solution. The HER performances of *fcc*‐Ag nanoparticles (NPs), *fcc*‐Au NSs, and the commercial Pt/C catalyst were also measured under identical conditions for comparison. As shown in **Figure** **4**a, the *fcc*‐Au NSs (Figure S7, Supporting Information) and *fcc*‐Ag NPs (Figure S8, Supporting Information) exhibit negligible HER activity in the investigated potential window. In contrast, the ladder‐like Au@PtAg nanoframes show superior HER activity. At the optimized loading amount of 0.025 mg_Pt_ cm^−2^ (Figure S9, Supporting Information), the polarization curve of the ladder‐like Au@PtAg nanoframes is nearly overlapped with that of the commercial Pt/C catalyst (Figure 4a), suggesting their comparable HER performances. Specifically, a small Tafel slope of the ladder‐like Au@PtAg nanoframes (28.3 mV dec^−1^) is also comparable to that of Pt/C (27.3 mV dec^−1^) (Figure 4b). It indicates that the Volmer‐Tafel mechanism is involved in the HER processes of both ladder‐like Au@PtAg nanoframes and Pt/C.^[^ ^33^, ^34^ ^]^ At the current density of 10 mA cm^−2^, the overpotential of ladder‐like Au@PtAg nanoframes is only −16.4 mV, while Pt/C requires the overpotential of −18.5 mV (left panel in Figure 4c). All the aforementioned results suggest the superior HER activity of the ladder‐like Au@PtAg nanoframes in the acid solution. Moreover, through the Cu underpotential deposition method, the electrochemically active surface area (ECSA) of the ladder‐like Au@PtAg nanoframes was measured to be 80.4 m^2^ g^−1^, which is much higher than that of Pt/C (54.8 m^2^ g^−1^) (right panel in Figure 4c, and Figure S10, Supporting Information). Such high ECSA of the ladder‐like Au@PtAg nanoframes could be attributed to their hollow structure which is beneficial to the exposure of more active sites.^[^ ^35^, ^36^ ^]^ Furthermore, the turnover frequency (TOF) values of the ladder‐like Au@PtAg nanoframes and Pt/C were calculated on the basis of the corresponding estimated numbers of active sites. As shown in Figure S11 (Supporting Information), the ladder‐like Au@PtAg nanoframes exhibit a TOF value of 9.3 H_2_ s^−1^ at the overpotential of ‐40 mV. The aforementioned results suggest that the high HER activity of the ladder‐like Au@PtAg nanoframes could be attributed to their high intrinsic activity and the large ECSA from their hollow structures. Additionally, the electrocatalytic stability of ladder‐like Au@PtAg nanoframes for HER was also investigated by an accelerated duration test. As shown in Figure 4d, almost no shift of the polarization curve was observed after 10000 potential cycling between 0.1 to −0.1 V (vs reversible hydrogen electrode (RHE)). After the durability test, we further characterized the ladder‐like Au@PtAg nanoframes, and the ladder‐like hollow structure of the Au@PtAg nanoframes was maintained (Figure S12, Supporting Information). These results clearly indicate the high electrocatalytic and structural stability of the ladder‐like Au@PtAg nanoframes toward HER in the acidic solution.

## 3 Conclusion

In summary, the effect of the crystal phase in the selective etching of Au has been systematically studied. The coating of Pt and Ag together with the crystal‐phase‐selective etching in the 4H/*fcc* heterophase Au NWs result in the formation of ladder‐like Au@PtAg nanoframes with new morphology and hollow structure. The developed etching method has also been used to realize the selective etching of 2H‐Au in the *fcc*‐2H‐*fcc* Au NRs and 2H/*fcc* Au NSs to obtain the dimer‐like Au@PtAg nanoframes and 2D Au@PtAg nanoframes, respectively. As a proof‐of‐concept application, the obtained ladder‐like Au@PtAg nanoframes were used as an electrocatalyst for HER in acidic media, exhibiting superior electrocatalytic activity. These findings reveal the significance of phase engineering in the controlled synthesis of novel multimetallic nanoframes with hollow architectures via the selective etching approach. We believe that our strategy can open up new way for the preparation of novel and complicated hollow nanomaterials with desired composition, crystal phase, and morphology to tailor their physicochemical properties for various applications.

## Conflict of Interest

The authors declare no conflict of interest.

## Supporting information

Supporting Information

## Data Availability

The data that support the findings of this study are available from the corresponding author upon reasonable request.
