# Supplementary material for: Crystal‐Phase‐Selective Etching of Heterophase Au Nanostructures
Source: Small Methods. 2024 Jul 6;8(11):2400430. doi: 10.1002/smtd.202400430 (PMC11579570; doi:10.1002/smtd.202400430)
Supplement: Supplementary file 1 — Supporting Information [file SMTD-8-2400430-s001.pdf]

small
methods

Supporting Information

for *Small Methods*, DOI 10.1002/smtd.202400430

Crystal-Phase-Selective Etching of Heterophase Au Nanostructures

*Faisal Saleem, Guangyao Liu, Guigao Liu, Bo Chen, Qinbai Yun, Yiyao Ge, An Zhang, Xixi Wang, Xichen Zhou, Gang Wang, Lingwen Liao, Zhen He, Lujiang Li and Hua Zhang**

Supporting Information

Crystal-Phase-Selective Etching of Heterophase Au Nanostructures

Faisal Saleem[1,2†], Guangyao Liu[1†], Guigao Liu[1,3†], Bo Chen[1,4†], Qinbai Yun[1], Yiyao Ge[1], An Zhang[1], Xixi Wang[1], Xichen Zhou[1], Gang Wang[5], Lingwen Liao[1,6], Zhen He[1,7], Lujiang Li[1], and Hua Zhang[1,7,8,9*]

[1]Department of Chemistry, City University of Hong Kong, Hong Kong, China

[2]Key Laboratory of Flexible Electronics (KLOFE) & Institute of Advanced Materials (IAM) & School of Flexible Electronics (Future Technologies), Nanjing Tech University, Nanjing, 211816, China

[3]National Special Superfine Powder Engineering Research Center, School of Chemistry and Chemical Engineering, Nanjing University of Science and Technology, Nanjing, 210094, China

[4]State Key Laboratory of Organic Electronics and Information Displays & Jiangsu Key Laboratory for Biosensors, Institute of Advanced Materials, Nanjing University of Posts and Telecommunications, Nanjing 210023, China

[5]Department of Chemistry, The Chinese University of Hong Kong, Hong Kong, China

[6]Key Laboratory of Materials Physics, Anhui Key Laboratory of Nanomaterials and Nanotechnology, Institute of Solid State Physics, Chinese Academy of Sciences, Hefei, 230031, China

[7]Hong Kong Institute for Clean Energy, City University of Hong Kong, Kowloon, Hong Kong, China

[8]Hong Kong Branch of National Precious Metals Material Engineering Research Center (NPMM), City University of Hong Kong, Hong Kong, China

[9]Shenzhen Research Institute, City University of Hong Kong, Shenzhen, 518057, China

[†]These authors contributed equally to this work.

*Correspondence to: hua.zhang@cityu.edu.hk

Chemicals

Gold(III) chloride hydrate ($HAuCl_4$, ∼50% Au basis), hexane (99%), potassium tetrachloroaurate ($KAuCl_4$, 99.995%), 1,2-dichloropropane (99%), 1,2-hexadecanediol (90%), oleylamine (70%), toluene (99.8%), platinum (II) acetylacetonate (97%), tris(hydroxymethyl) aminomethane (99.8%), silver nitrate (99%), potassium iodide (99%), polyvinylpyrrolidone (PVP, Mw = 10K), polyvinylpyrrolidone (PVP, Mw = 58K), formamide (99.5%) and other chemicals do not mention here were purchased from Sigma-Aldrich. Ethanol (absolute, 99.9%) was purchased from Merck (Nordic European Centre, Singapore). All the chemical reagents were used as received without any further purification. All aqueous solutions were prepared using Milli-Q water with a resistivity of 18.2 MΩ·cm at room temperature.

Characterizations

Transmission electron microscopy (TEM) images, high-resolution TEM (HRTEM) images, and selected area electron diffraction (SAED) patterns were taken on the JEOL JEM-2100F (JEOL, Tokyo, Japan) microscope operated at 200 kV. The high-angle annular dark-field scanning TEM (HAADF-STEM) images were taken on a JEOL ARM200F (JEOL, Tokyo, Japan) spherical aberration-corrected transmission electron microscope operated at 200 kV. Energy dispersive X-ray spectroscopy (EDS) data were obtained by using the JEOL JEM-2100F and JEOL ARM200F microscopes. X-ray diffraction (XRD) patterns were recorded with a Rigaku SmartLab X-ray diffractometer, using a Cu K_α radiation source (λ=1.5406 Å). The inductively coupled plasma optical emission spectroscopy (ICP-OES) was performed on the Optima 8000 DV ICP-OES system.

Methods

Synthesis of 4H/face-centered cubic (*fcc*) Au nanowires (NWs).

The 4H/*fcc* Au NWs were prepared by using our previously reported method.[1]

Synthesis of *fcc*-2H-*fcc* Au nanorods (NRs).

The *fcc*-2H-*fcc* Au NRs were prepared by using our previously reported method.[2]

Synthesis of 2H/*fcc* Au nanosheets (NSs)

The 2H/*fcc* Au NSs were prepared by using our previously reported method.[3]

Preparation of gel-like material as the precursor for the etchant solution

The gel-like material was prepared by using our previously reported method with slight modifications.[4] In a typical synthesis, a mixture of PVP (600 mg, Mw = 10K), tris(hydroxymethyl)aminomethane (80 mg), and formaldehyde solution (4 mL) was transferred into a 25 mL Teflon-lined stainless-steel autoclave, which was kept at 190 ^{o}C for 3 h. After cooling to room temperature, acetone (20 mL) was added to the obtained solution, which was then centrifuged at 8,000 rpm for 5 min. The brownish gel-like material was collected and dried at 90 ^{o}C for 15 min.

Synthesis of *fcc*-Au NSs

The *fcc*-Au NSs were prepared by using the aforementioned gel-like material as one of the precursors. Typically, the gel-like material was dissolved in formamide (4 mL) followed by the addition of $HAuCl_4$ (8 mg) and KI (60 mg). The obtained mixture solution was transferred into a 25 mL Teflon-lined stainless-steel autoclave, which was held at 130 ^{o}C for 3 h. After cooling the autoclave to room temperature, the obtained product was collected by centrifugation at 10,000 rpm for 5 min and washed with ethanol for 3 times.

Synthesis of *fcc*-Ag nanoparticles (NPs)

$AgNO_3$ (20 mg), PVP (200 mg, Mw = 58K) and formamide (5 mL) were sequentially added in a 12 mL glass vial, followed by ultrasonicating for 5 min. After the color of the solution changed to red, the solution was kept at room temperature for 12 h. the obtained product was collected by centrifugation at 14,000 rpm for 5 min and washed with ethanol for 3 times.

Ligand exchange

Ligand exchange of heterophase Au nanomaterials was conducted by following the previously reported method with some modifications.[5] The aforementioned 4H/*fcc* Au NWs, *fcc*-2H-*fcc* Au NRs, or 2H/*fcc* Au NSs were re-dispersed in 20 ml of chloroform with a concentration of 0.5 mg mL^{-1}

determined by ICP-OES, followed by the addition of PVP (500 mg, Mw = 58K). After vigorous stirring for three days, the PVP-capped products were precipitated by adding 20 mL of ethanol and collected by centrifugation at 6,500 rpm for 5 min. The sample was washed 3 times with ethanol to remove the excess PVP and finally re-dispersed in ethanol.

Selective etching of heterophase Au nanomaterials

The etching process is schematically shown in Figure S1. First, the aforementioned gel-like material was dissolved in formamide (4 mL) followed by the addition of $Pt(acac)_2$ (7.8 mg), $AgNO_3$ (4.3 mg), and KI (60 mg) to prepare the etchant solution. Subsequently, 0.3 mg of pre-synthesized 4H/*fcc* Au NWs, *fcc*-2H-*fcc* Au NRs, or 2H/*fcc* Au NSs were re-dispersed in the aforementioned etchant solution. After sonicated for 20 min, a homogeneous mixture was obtained for the selective etching of Au. Subsequently, 0.05 mL of the obtained solution was divided into 5 drops and transferred into a 25 mL Teflon-lined stainless-steel autoclave, which was maintained at 130 ^{o}C for 3 h. After the autoclave cooled down naturally, the products were collected by centrifugation at 14,000 rpm for 4 min and then washed with the mixture of ethanol and acetone (v:v=4:1) for 3 times.

Electrochemical measurements

Electrochemical hydrogen evolution reaction (HER) measurements were conducted on the Autolab workstation (PGSTAT12) at room temperature. Glassy carbon electrode with a diameter of 3 mm coated with catalysts was used as the working electrode. The graphite rod and Ag/AgCl (3 M KCl) were used as counter electrode and reference electrode, respectively. The Ag/AgCl electrode was calibrated with respect to a reversible hydrogen electrode (RHE). The working electrodes were prepared by drop-casting appropriate amounts of the catalyst inks onto the glassy carbon electrodes. The loading amounts of catalysts were determined by ICP-OES (**Table S1**). Using the same procedure, the working electrodes of Ag NPs, Au NSs, and the commercial Pt/C catalyst were also prepared. The loading amounts of Pt were kept to be 0.025 mg cm^{-2}.

The HER test was carried out in N_2-saturated 0.5 M H_2SO_4 aqueous solution. The polarization curves were recorded at a scanning rate of 5 mV s^{-1}. The accelerated durability tests were performed at room

temperature in N_2-saturated 0.5 M H_2SO_4 aqueous solution by applying the cyclic potential sweeps between 0.066 V and -0.134 V (vs. reversible hydrogen electrode (RHE)) at a scan rate of 100 mV s^{-1} for 10,000 cycles. After potential cycling, the polarization curve was measured at a scanning rate of 5 mV s^{-1}. The current density for HER was normalized by the geometric surface area of the electrode.

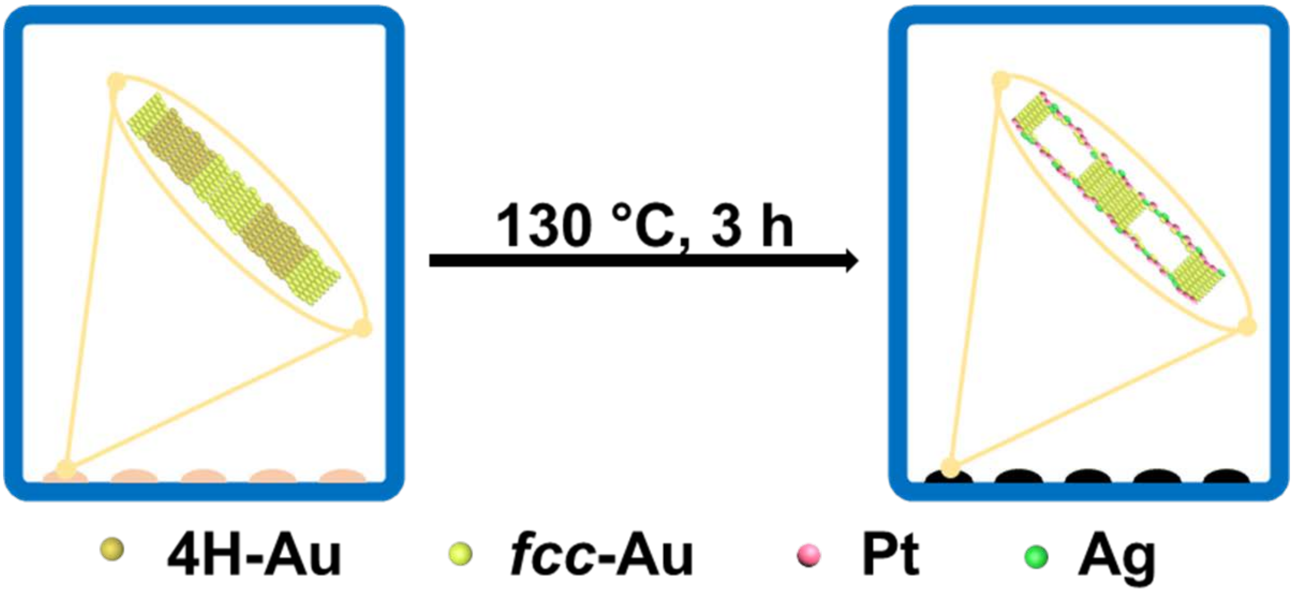

Figure S1. Schematic illustration of the crystal-phase-selective etching of 4H/*fcc* heterophase Au NW to form ladder-like Au@PtAg nanoframe.

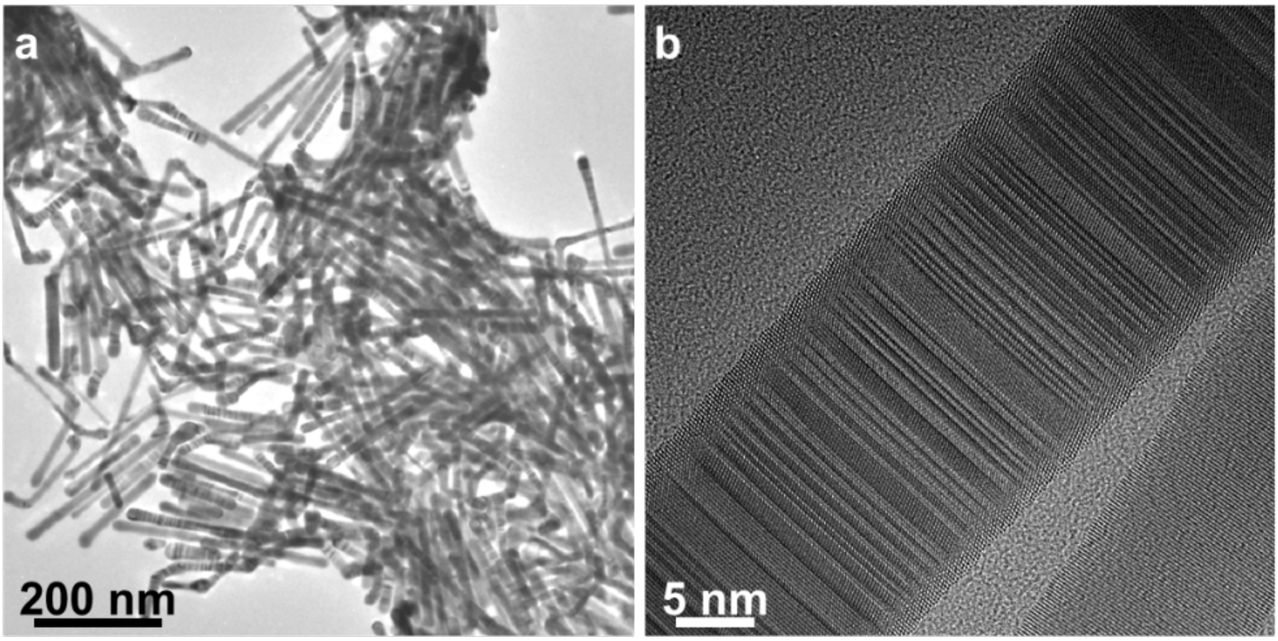

Figure S2. Characterization of as-synthesized 4H/*fcc* Au NWs. a) Low-magnification TEM image of 4H/*fcc* Au NWs. b) HRTEM image of a representative 4H/*fcc* Au NW.

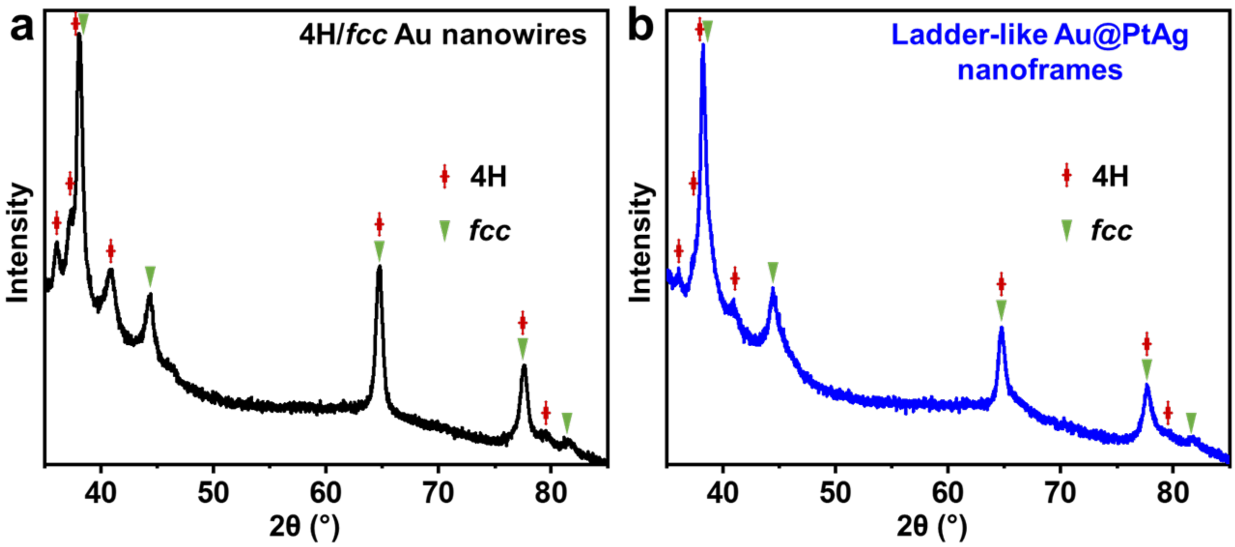

Figure S3. a, b) XRD patterns of the original 4H/*fcc* Au NWs (a) and the ladder-like Au@PtAg nanoframes (b).

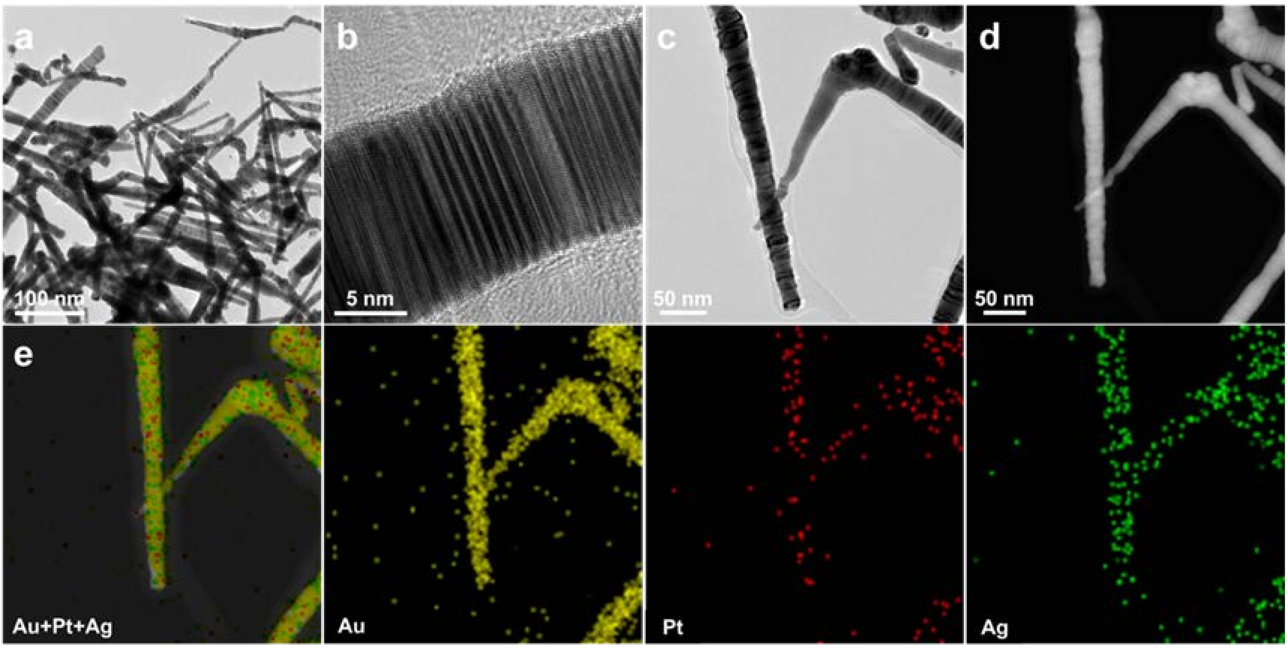

Figure S4. Characterization of as-synthesized core-shell Au@PtAg nanorods. a) Low-magnification TEM image, b) HRTEM image, c) bright-field HRTEM image and d) dark-field STEM image of Au@PtAg core-shell nanorods. e) The corresponding EDS elemental mapping images of the Au@PtAg core-shell nanorods.

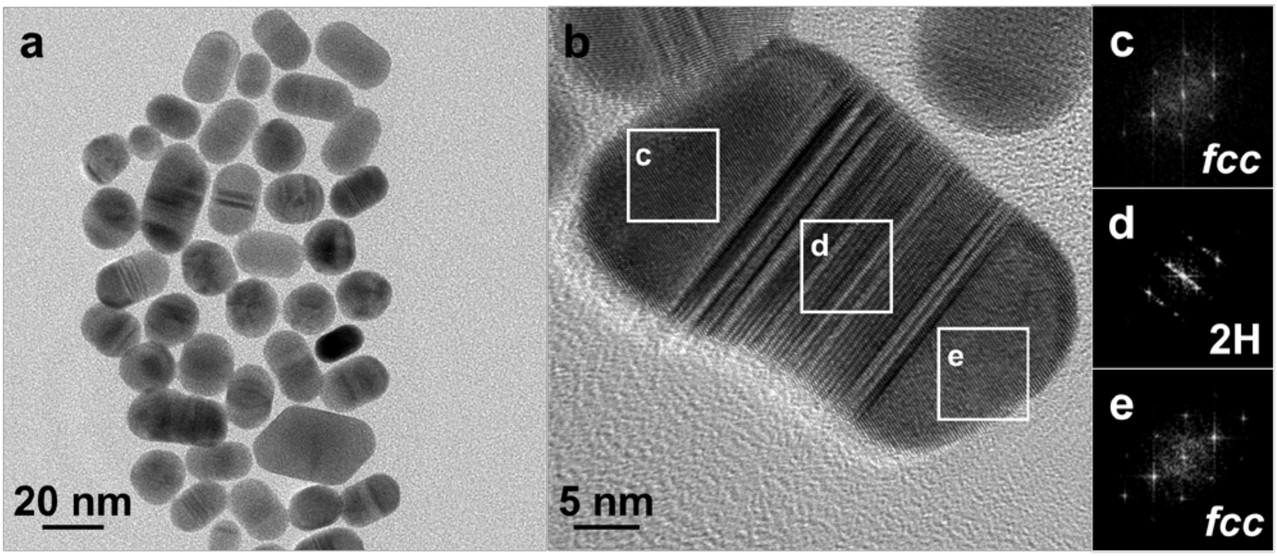

Figure S5. Characterization of *fcc*-2H-*fcc* Au NRs. a) TEM image. b) HRTEM image. c-e) The corresponding fast Fourier transform (FFT) patterns taken from the selected areas in (b).

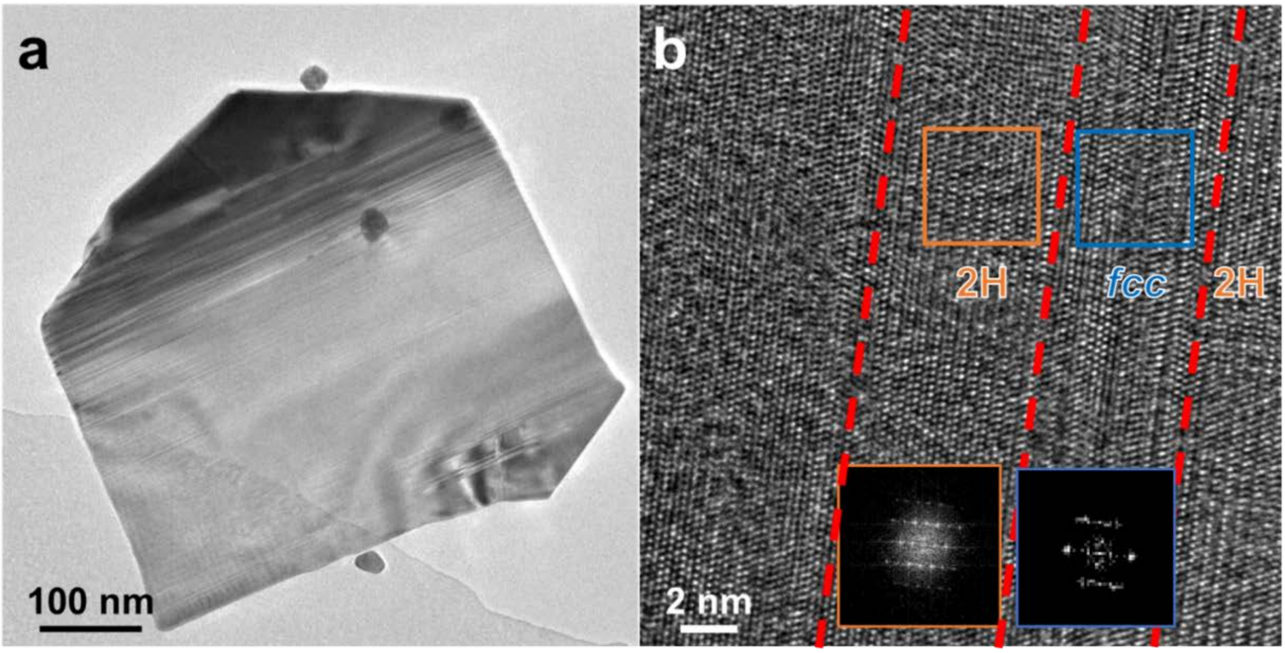

Figure S6. Characterization of 2H/*fcc* Au NSs. a) TEM image. b) HRTEM image. Insets: the corresponding FFT patterns of the selected areas.

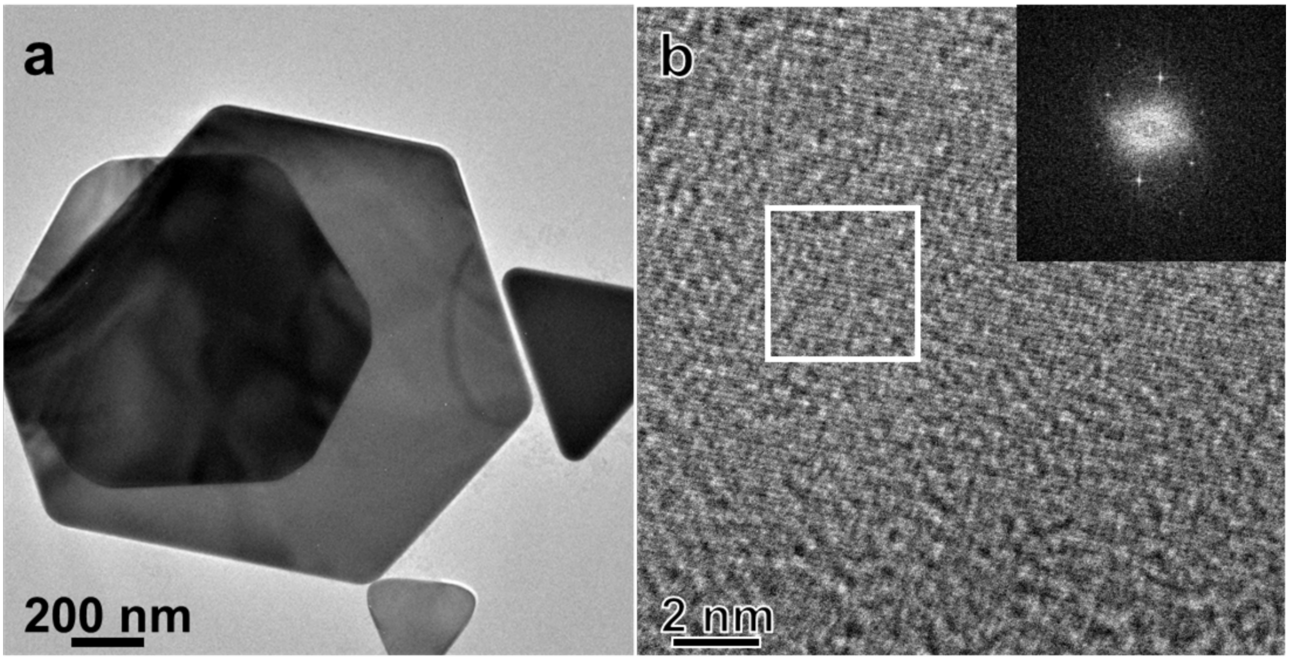

Figure S7. Characterization of *fcc*-Au NSs. a) TEM image. b) HRTEM image. Inset: the corresponding FFT pattern.

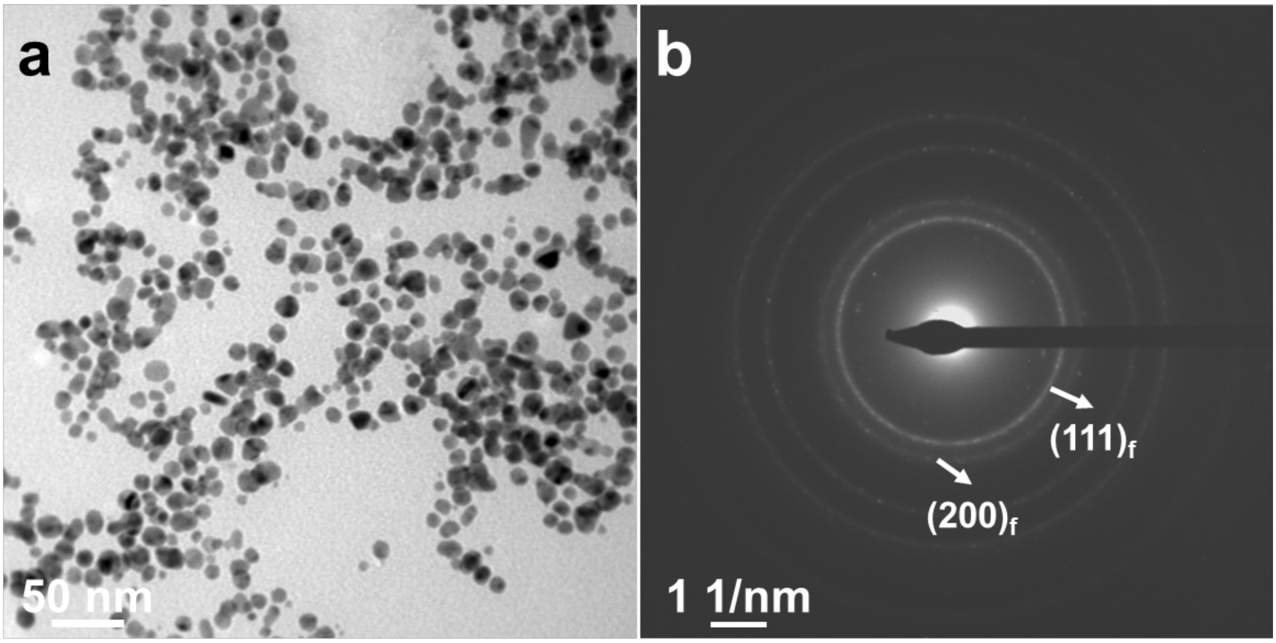

Figure S8. Characterization of *fcc*-Ag NPs. a) TEM image. b) SAED pattern.

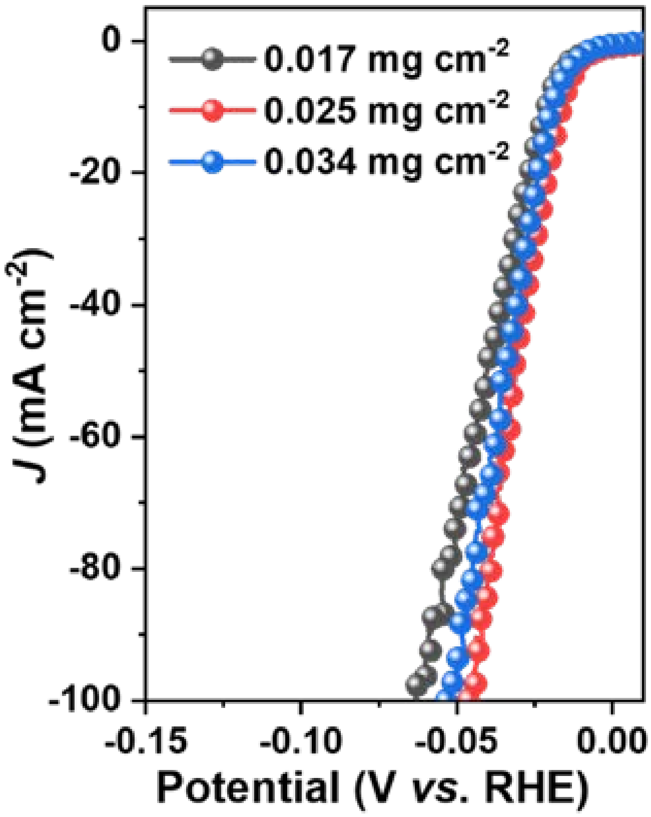

Figure S9. HER polarization curves of the ladder-like Au@PtAg nanoframes with three different loadings of Pt in 0.5 M H_2SO_4 aqueous solution.

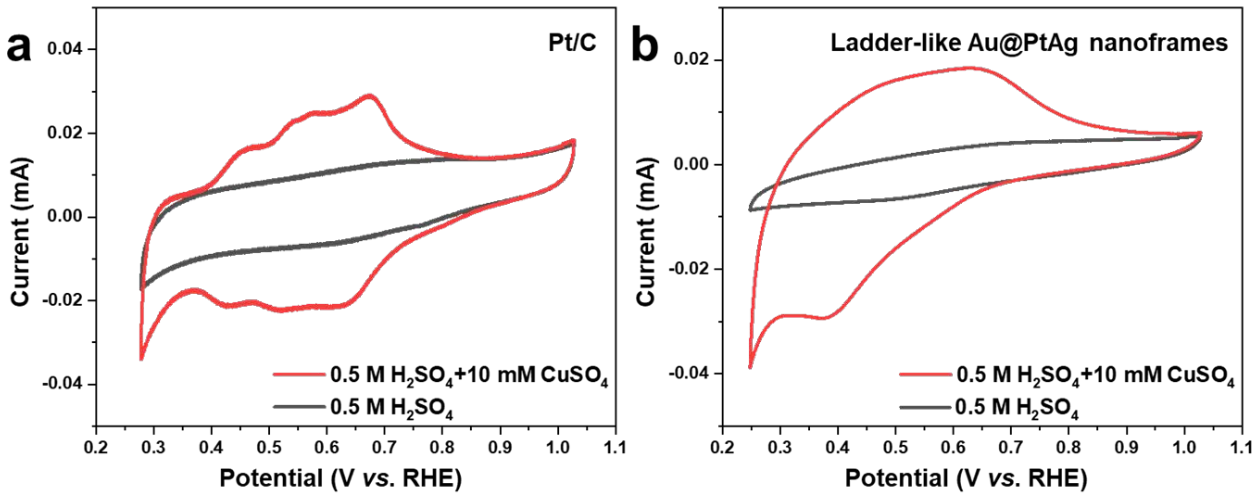

Figure S10. Calculation of the number of active sites and the electrochemically active surface area (ECSA) by using the Cu underpotential deposition (UPD) method. a, b) Cyclic voltammetry curves of Pt/C (a) and the ladder-like Au@PtAg nanoframes (b), recorded at a scanning rate of 10 mV s^{-1} in N_2-saturated 0.5 M H_2SO_4 in the presence and absence of 10 mM $CuSO_4$.

The Cu UPD is a method widely used for quantifying the number of active sites and the ECSA of catalysts.[6] By using this method, the number of active sites was calculated based on the Cu UPD stripping charge (Q_{Cu}, $Cu_{upd} \rightarrow Cu^{2+} + 2e^-$) with the following equation:

$$n = Q_{Cu}/(2Fm)$$

where F is the Faraday constant (96485 C mol^{-1}), and m is the Pt mass loading (1.8×10^{-6} g).

The turnover frequency (TOF, H_2 s^{-1}) can be calculated based on the following equation:

$$TOF = I/(2Fnm)$$

where I is the current (A) during the LSV measurement. The factor 2 is the number of electrons transferred, since two electrons are required to form one H_2 molecule.

Assuming a value of 420 μC cm^{-2} for a saturated Cu_{upd} monolayer formation on active metal sites, the ECSA can be calibrated as follows:

$$ECSA = Q_{Cu}/(m \times 420\ \mu C\ cm^{-2})$$

The number of active sites and the ECSA of the ladder-like Au@PtAg nanoframes were calculated to be 1.75×10^{-3} mol g^{-1} and 80.4 m^2 g^{-1}, respectively. The number of active sites and the ECSA of Pt/C were calculated to be 1.19×10^{-3} mol g^{-1} and 54.8 m^2 g^{-1}, respectively.

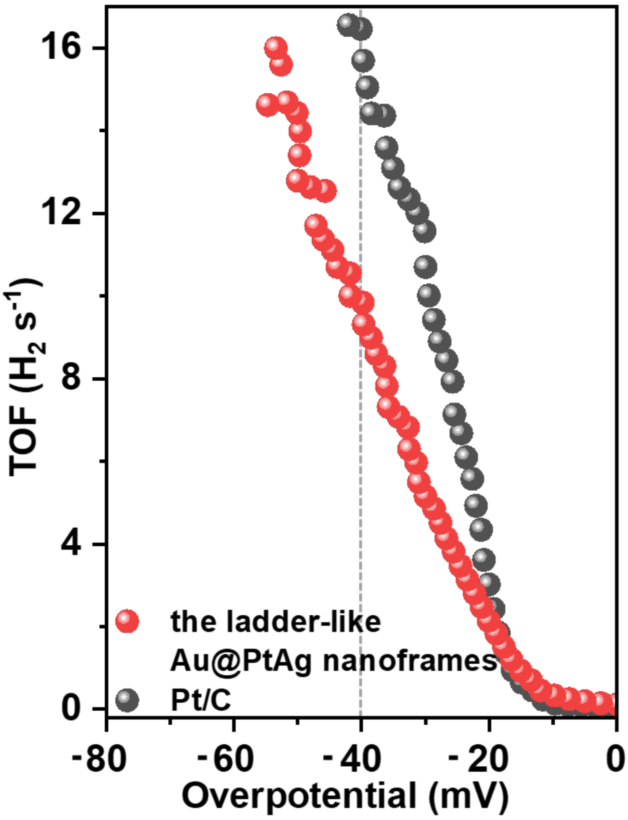

Figure S11. Comparison of the TOF values of the ladder-like Au@PtAg nanoframes and commercial Pt/C.

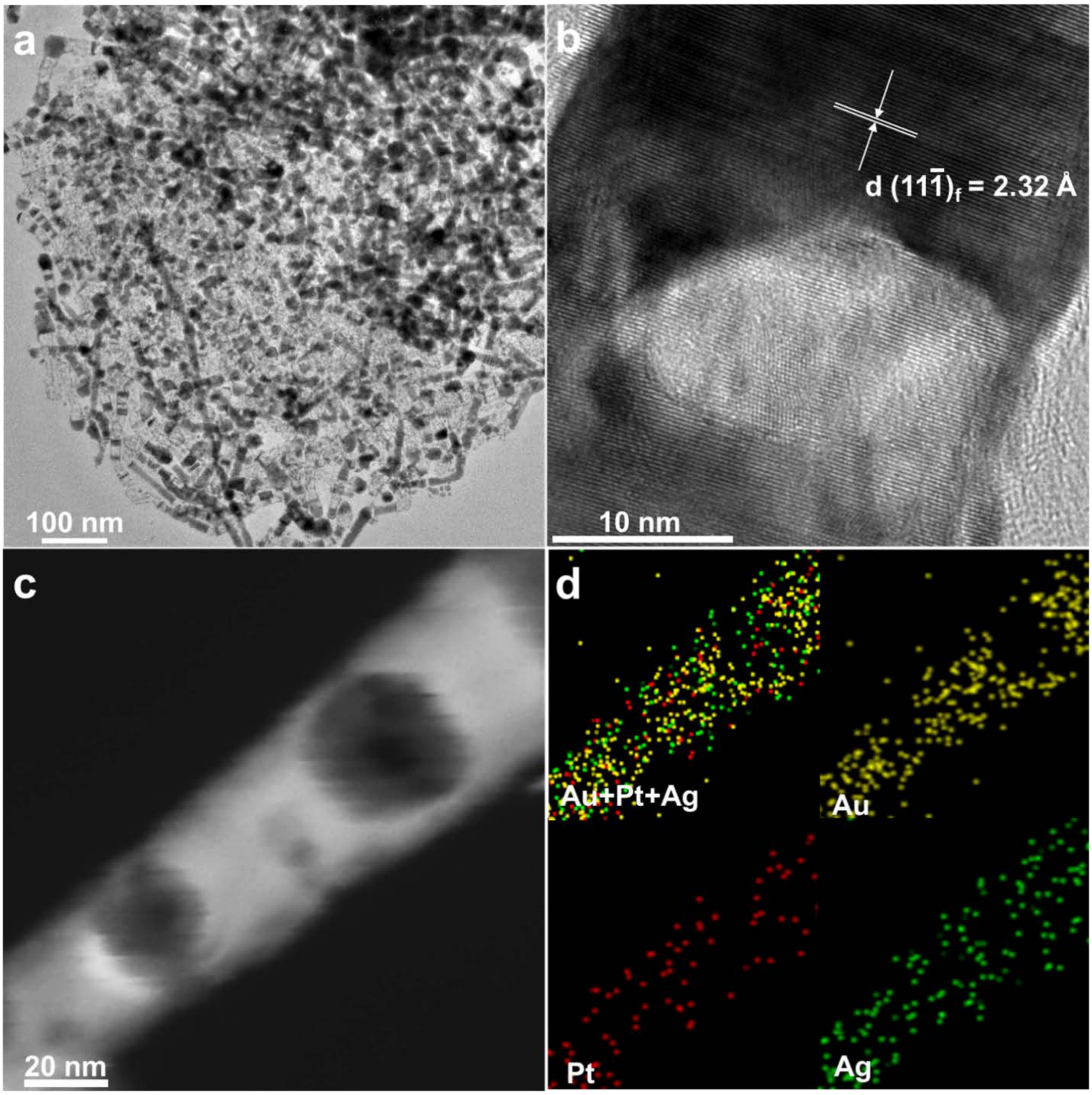

Figure S12. Characterization of the ladder-like Au@PtAg nanoframes after the electrocatalytic stability test. a) Low-magnification TEM image. b) HRTEM image. c) Dark-field STEM image. d) Corresponding EDS elemental mappings.

Table S1. ICP-OES characterizations showing the atomic ratios of Au, Pt and Ag of the ladder-like Au@PtAg nanoframes, dimer-like Au@PtAg nanoframes and the 2D Au@PtAg nanoframes.

ICP-OES results	Ladder-like Au@PtAg nanoframes	Dimer-like Au@PtAg nanoframes	2D Au@PtAg nanoframes
Au	57.4	55.0	77.9
Pt	9.0	15.6	6.1
Ag	33.6	29.4	16.0

Reference

[1] Y. Chen, Z. Fan, Z. Luo, X. Liu, Z. Lai, B. Li, Y. Zong, L. Gu, H. Zhang, *Adv. Mater.* **2017**, *29*, 1701331.

[2] Z. Fan, M. Bosman, Z. Huang, Y. Chen, C. Ling, L. Wu, Y. A. Akimov, R. Laskowski, B. Chen, P. Ercius, J. Zhang, X. Qi, M. H. Goh, Y. Ge, Z. Zhang, W. Niu, J. Wang, H. Zheng, H. Zhang, *Nat. Commun.* **2020**, *11*, 3293.

[3] J. Liu, W. Niu, G. Liu, B. Chen, J. Huang, H. Cheng, D. Hu, J. Wang, Q. Liu, J. Ge, P. Yin, F. Meng, Q. Zhang, L. Gu, Q. Lu, H. Zhang, *J. Am. Chem. Soc.* **2021**, *143*, 4387.

[4] F. Saleem, Z. Zhang, X. Cui, Y. Gong, B. Chen, Z. Lai, Q. Yun, L. Gu, H. Zhang, *J. Am. Chem. Soc.* **2019**, *141*, 14496.

[5] G. Lu, S. Li, Z. Guo, O. K. Farha, B. G. Hauser, X. Qi, Y. Wang, X. Wang, S. Han, X. Liu, J. S. DuChene, H. Zhang, Q. Zhang, X. Chen, J. Ma, S. C. J. Loo, W. D. Wei, Y. Yang, J. T. Hupp, F. Huo, *Nat. Chem.* **2012**, *4*, 310.

[6] C. L. Green, A. Kucernak, *J. Phys. Chem. B* **2002**, *106*, 1036.
